# Periodontitis and Mild Cognitive Impairment Risk in Diabetic Patients: Insights from an Exploratory Analysis

**DOI:** 10.3390/dj13110505

**Published:** 2025-11-03

**Authors:** Aulia Ramadhani, Kumiko Minagawa, Sachiko Takehara, Noboru Kaneko, Takaho Yamada, Masaru Kitazawa, Hirohito Sone, Yusran Ady Fitrah, Kaname Nohno, Hiroshi Ogawa

**Affiliations:** 1 Division of Preventive Dentistry, Graduate School of Medical and Dental Sciences, Niigata University, Niigata 951-8514, Japan; aulia.ramadhani@fkg.unair.ac.id (A.R.); sakairi@dent.niigata-u.ac.jp (K.M.); takeh@dent.niigata-u.ac.jp (S.T.); nkaneko@dent.niigata-u.ac.jp (N.K.); 2 Division of Dental Public Health, Department of Basic Dental Sciences and Dental Public Health, Faculty of Dental Medicine, Universitas Airlangga, Surabaya 60132, Indonesia; 3 Department of Hematology, Endocrinology and Metabolism, Faculty of Medicine, Niigata University, Niigata 951-8510, Japan; t-yamada@med.niigata-u.ac.jp (T.Y.); m-kitazawa@med.niigata-u.ac.jp (M.K.); sone@med.niigata-u.ac.jp (H.S.); 4 Department of Neurology, Faculty of Medicine, Hasanuddin University, Makassar 90245, Indonesia; yusranadyf@med.unhas.ac.id; 5 Department of Oral Health and Welfare, Graduate School of Medical and Dental Sciences, Niigata University, Niigata 951-8514, Japan; no2@dent.niigata-u.ac.jp

**Keywords:** periodontitis, diabetes mellitus, cognitive dysfunction, inflammation, health risk

## Abstract

**Background:** Cognitive impairment, including dementia, is a growing global health challenge, particularly as populations age. Previous studies have identified periodontitis and diabetes mellitus as modifiable risk factors for dementia, potentially linked through systemic inflammation. We hypothesize that systemic inflammation induced by periodontitis may contribute to an increased risk of cognitive impairment. Therefore, this study aims to explore the correlation between periodontal inflammation and the risk of Mild Cognitive Impairment (MCI) in type II diabetes mellitus. **Materials and Methods:** Baseline data analysis was performed as an analytical cross-sectional study among diabetic patients aged 40 and older who met the inclusion criteria from a randomized controlled trial (RCT) from November 2020 to April 2023. Periodontal inflammation was measured using the Periodontal Inflamed Surface Area (PISA) score. The MCI risk score was calculated using blood samples to assess eight protein markers correlated to MCI (ApoA1, TTR, C3, Albumin, ApoC1, A1BG, A2AP, and HPX). Fisher’s exact test and Spearman’s correlation analysis were performed. **Results:** 29 T2DM patients were included in the study. There was a significant difference in MCI risk score between the low and high PISA levels group (*p* < 0.05). Patients with low PISA scores tend to have a lower risk of MCI (*p* < 0.00). Variables correlated with MCI risk are PISA (ρ = 0.37; *p* < 0.05) and TTR levels (ρ = −0.51; *p* < 0.01). ApoA1 has a correlation with CRP (ρ = 0.42; *p* < 0.05) and IL-6 (ρ = 0.43; *p* < 0.05), and C3 (ρ = 0.42; *p* < 0.05) was correlated with CRP. **Conclusions:** This study found that periodontal inflammation status has a potential correlation to the risk of MCI.

## 1. Introduction

Dementia, as a syndrome of cognitive impairment, represents a significant global public health challenge, particularly in aging populations. Current reports indicate that more than 55 million people worldwide are affected by dementia, with approximately 10 million new cases diagnosed annually. Beyond being the seventh leading cause of death, dementia contributes substantially to disability and dependency among those affected [1,2]. It is characterized by a decline in cognitive functions that exceeds normal age-related changes [2]. Various risk factors have been identified, including age, genetic predisposition, and pathological conditions, such as diabetes mellitus and periodontitis [3].

Type II Diabetes Mellitus (T2DM) is an important risk factor for dementia and shares a bidirectional relationship with periodontitis [4,5]. Previous studies have reported a higher risk of developing dementia in older diabetic patients with periodontitis [5,6,7]. Both T2DM and periodontitis are global health concerns with significant implications for human well-being and health expenditure [8,9]. Notably, both conditions may contribute to neurodegeneration through systemic inflammation [10,11].

Given that primary degenerative dementias result from progressive, irreversible neural destruction, early symptom management is essential. This management involves addressing modifiable risk factors of cognitive impairment, such as periodontal and systemic health. Mild cognitive impairment (MCI), which serves as the preclinical and transitional stage between normal aging and dementia [12], is viewed as a strategic target for interventions aimed at delaying the progression to dementia [13]. While the relationship between periodontitis, diabetes mellitus, and cognitive impairment has been explored in previous studies, the specific impact of systemic inflammation exacerbated by periodontitis or diabetes on the MCI risk remains unclear. To date, no study has specifically assessed inflammation levels in periodontal tissues using the Periodontal Inflamed Surface Area (PISA) and linked them to the risk of developing MCI. Early screening, prevention, and targeted intervention in individuals without a history of cognitive disorders are essential to reduce the likelihood of severe neurodegenerative diseases and prevent progression to severe neurodegenerative diseases.

This study addressed this gap by evaluating inflammation marker levels in diabetic patients with periodontitis and comparing the risk of MCI between those with low and high levels of periodontal inflammation. We hypothesize that diabetic patients with higher levels of periodontal inflammation are at greater risk for MCI. Thus, this study aims to investigate the relationship between periodontal inflammation and MCI risk in diabetic patients and to explore the associations between each biomarker.

## 2. Materials and Methods

### 2.1. Study Design

This study followed an analytical cross-sectional study design and involved the analysis of baseline data from a randomized controlled trial (RCT) from a previously conducted intervention study at Niigata University. The original study primarily investigated the effect of antibiotic periodontal therapy on cerebral infarction risk in patients with diabetes. In addition to cerebral infarction risk, the baseline data included measurements of Mild Cognitive Impairment (MCI) risk using blood-based biomarkers and periodontal inflammation assessed by the Periodontal Inflamed Surface Area (PISA) score. This secondary analysis aims to explore the association between periodontal inflammation and MCI risk in the same patient population. The original study was approved by the Ethics Committee of Niigata University (Approval Numbers: 2020-0126 and 2023-0048; approval date: 19 August 2020) and registered in the University Hospital Medical Information Network (UMIN) Center under trial number UMIN000042458.

For the current analysis, participants were re-grouped based on the score of PISA, which represents the degree of periodontal inflammation. This grouping was selected to explore the levels of MCI risk blood-based biomarkers in different categories of periodontal inflammation status and investigate their potential relationship.

### 2.2. Study Participants

The data includes thirty patients with type 2 diabetes mellitus (T2DM) and periodontitis who were recruited and signed the written informed consent by the Department of Endocrinology at Niigata University Hospital from November 2020 to April 2023.

#### 2.2.1. Inclusion Criteria

Patients were eligible for inclusion if they met the following conditions: age ≥ 40 years; HbA1c ≥ 6.0%; no history of insulin therapy; maintenance of a stable diabetes treatment regimen for at least the preceding 2 months; no history of stroke; non-smoker status; and presence of at least 10 teeth with ≥4 periodontal pockets measuring ≥4 mm in depth.

#### 2.2.2. Exclusion Criteria

Patients were excluded if they had HbA1c < 6.0%, had undergone a change in diabetes treatment within the past 2 months, or had a history of cerebral infarction.

### 2.3. Data Collection and Variables

In this secondary analysis, only baseline data from the dataset were utilized. Data analysis includes general health variables, periodontal health, inflammation status, and MCI risk. The authors had access to data containing identifiable participant information during data collection. However, all data were anonymized before analysis in this secondary study to ensure confidentiality.

#### 2.3.1. General Health Variables

General health variables, including respondent characteristics (age and gender), Body Mass Index (BMI), and blood glucose (HbA1c), were collected from the patients.

#### 2.3.2. Periodontal Health

Periodontal health data used in this analysis include the number of teeth, the percentage of sites with Probing Pocket Depth (PPD) more than 4 mm, Clinical Attachment Level (CAL), Bleeding on Probing (BOP), and Periodontal Inflamed Surface Area (PISA) [14]. Periodontal health data collection was conducted at the Department of Preventive Dentistry, Niigata University Hospital, Niigata, Japan, by a single examiner. The single examiner has been calibrated prior to the study according to the American Academy of Periodontology (AAP) guidelines. Probing was carried out at six points per tooth for all present teeth, applying a force of approximately 20 to 25 g, with measurements rounded to the nearest millimeter.

#### 2.3.3. Inflammation Status and MCI Risk

The dataset includes blood sample analysis, such as inflammation-related markers (Interleukin-6 (IL-6), C-Reactive Protein (CRP), and Tumor Necrosis Factor-α (TNF-α)) and MCI risk through protein plasma analysis. The proteins involved in the analysis are the Amyloid-β sequester proteins (Apolipoprotein A1 (ApoA1), Complement C3, and Transthyretin (TTR)), the nutrition-related proteins (Albumin), lipid metabolism protein (Apolipoprotein C1 (ApoC1)), innate immunity and inflammation (Alpha-1-B-Glycoprotein (A1BG)), and coagulation-fibrinolytic protein (Alpha-2-Macroglobulin (A2M), Alpha-2-Anti Plasmin (A2AP), and Hemopexin (HPX)) [15,16]. The MCI risk score ranged from 0.00–0.50 (“No risk”), 0.51–1.00 (“Low risk”), 1.01–1.50 (“At risk”), and 1.51–2.00 (“High risk”) (MCI Screening Test Plus, MCBI Co, Ltd.,Tokyo, Japan) [17]. However, due to the limited number of patients, this study used only two ranges of score groups to categorize MCI risk: 0.00–1.00 (“No risk to low risk” of MCI) and 1.01–2.00 (“At risk to high risk” of MCI).

### 2.4. Statistical Analysis

Statistical analysis was performed using IBM SPSS version 28 (IBM Corp., Armonk, NY, USA). Due to the abnormal data distributions, a non-parametric test was applied in this study. The data were compared according to the PISA group. The Mann–Whitney U-test was used to compare the parameters between low and high PISA scores. Fisher’s exact test was performed to analyze the proportion of difference in the MCI risk between low and high PISA scores. Lastly, the Spearman correlation test was conducted to investigate the correlation between each variable. Values of *p* < 0.05 with 95% CI were considered significant. To enhance the robustness of the statistical estimates, we applied bootstrapping with 1000 resamples, drawn with replacement from the original dataset. The bootstrapped procedure was used to construct 95% confidence intervals for the correlation coefficient, allowing for a more reliable estimate of variability and accounting for potential non-normality in the data distribution [18].

## 3. Results

### 3.1. Patient Characteristics

Among thirty patients, the data of twenty-nine patients were analyzed. One patient was excluded due to missing data. Table 1 presents the patient’s characteristics by comparing demographic, health, periodontal status, inflammation status, and MCI risk parameters between the two groups categorized by PISA scores. The low PISA score group represented the patients with scores ≤ 300 in PISA, while the High PISA score group represented the patients with scores more than 300 in PISA. Results are presented as mean ± SD or n (%) and *p*-value of Mann–Whitney U-test.

Table 1 shows that 68.9% of the patients had low PISA scores, with 65% male. The average age was 61.60 in the low PISA score group and 67.11 in the high PISA score group. In general health data, BMI score and HbA1c level were observed. The HbA1c levels and BMI scores in the high PISA score group were slightly higher. However, no significant difference was observed (*p* < 0.05; CI 95%).

From the periodontal status, it was found that patients in both groups have 25 teeth on average. The low PISA score group has significantly better periodontal parameter scores, with lower PISA scores, probing depth, and number of sites with BOP (*p* < 0.01). The proportion of sites with probing depth ≥ 4 mm (PD ≥ 4 mm) is significantly higher in the >300 mm^2^ group (20.60 ± 18.37) than in the ≤300 mm^2^ group (6.73 ± 4.72) (*p* = 0.01).

The inflammation parameters (CRP, IL-6, and TNF- α) in the high PISA score group were higher, regardless of the significant difference. Meanwhile, three MCI risk parameters (C3, Hemopexin, and A2M) were higher in the high PISA score group. There were no significant differences observed.

Of the 29 patients screened for MCI risk, 13 patients (44.8%) were in the “At-risk to high-risk” groups. As much as 88.9% of patients in the high PISA score group were at risk to high risk of MCI. There was a significant difference in the mean value of MCI risk between low (0.69 ± 0.65) and high (1.37 ± 0.49) PISA score groups (*p* < 0.05). Patients in the high PISA score group possessed higher MCI risk scores.

### 3.2. The Risk of MCI in High PISA Scores Groups

According to Table 2, 88.9% of the patients had high PISA scores (>300 mm^2^) and are categorized as at-risk to high risk of MCI. The Fisher’s exact (CI 95%) test was performed to determine the correlation between PISA and MCI risk scores. An odds ratio of 24 indicates that those with high PISA scores are significantly more likely to be at “At-risk to high-risk” of MCI groups (*p* < 0.01).

**Figure 1:**
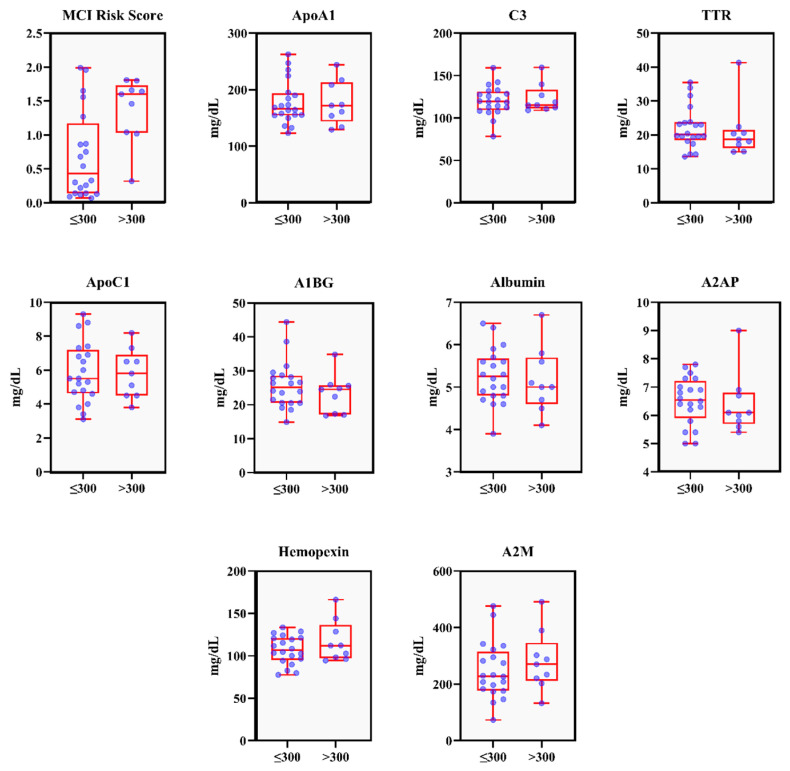
The distribution comparison of MCI risk biomarkers between the two PISA score groups presented in boxplots. Blue dots represent individual data points, and red boxes indicate the interquartile range (from the first to the third quartile), with whiskers and outliers shown. ApoA1: Apolipoprotein A1; C3: Complement C3; TTR: Transthyretin; A1BG: Alpha-1-B-Glycoprotein; ApoC1: Apolipoprotein C1; A2AP: Alpha-2-Antiplasmin; A2M: Alpha-2-Macroglobulin.

Figure 1 shows the distribution of individual values in both groups. The median MCI risk score appears to be higher in the high PISA group compared to the low PISA group. This suggests that individuals with a higher PISA score (>300) tend to have an average MCI risk score. Both groups have a few outliers, which may represent individuals with particularly high MCI risk scores relative to their group.

### 3.3. The Correlation Between Periodontal Status, Inflammation, and MCI Risk Markers

Table 3 presents the Spearman‘s correlation test result with 1000 samples bootstrapping using a 95% Confidence Interval. The Spearman’s correlation test assessed the relationship between each variable. According to the table, the MCI risk score has a positive correlation with PISA score (r = 0.37; *p* < 0.05), and the PISA was found to be negatively correlated with ApoC1 (r = −0.42; *p* < 0.05). Variables correlated with MCI risk are PISA (ρ = 0.37; *p* < 0.05) and TTR levels (ρ = −0.51; *p* < 0.01). CRP and IL-6, as the inflammation markers, were also found to be correlated with several MCI risk markers. CRP was correlated with ApoA1 (r = −0.42; *p* < 0.05), C3 (r = 0.42, *p* < 0.05), and HPX (r = 0.38; *p* < 0.05). Meanwhile, IL-6 was correlated with ApoA1 (r= −0.43; *p* < 0.05). However, although the *p*-value indicated significance, the bootstrapped 95% confidence interval for the correlation coefficient between CRP and HPX included zero ([lower bound, upper bound]), indicating potential variability in the estimate. Lastly, HbA1c as a diabetic marker did not correlate with any variables.

## 4. Discussion

This study investigated the correlation between periodontal inflammation and Mild Cognitive Impairment (MCI) risk using blood-based biomarkers in patients with type 2 diabetes mellitus (T2DM). Previously, systemic inflammation has been proposed as a key pathway linking diabetes, periodontal disease, and cognitive impairment, particularly among individuals with T2DM [19,20,21]. Studies by Rajendran (2024) and Sharma (2018) identified cognitive impairment in patients with diabetes mellitus and poor periodontal health, utilizing the Montreal Cognitive Assessment (MoCA) for cognitive evaluation [3,22]. Balasubramaniam (2022) further reported that a significant proportion of diabetic patients with chronic periodontitis had an increased risk for cognitive impairment, assessed with the Community Screening Instrument for Dementia (CSI-D) for evaluation [6].

The early detection of MCI and preventive interventions are crucial for slowing cognitive decline. Effective diagnostic biomarkers that facilitate timely and accurate diagnosis are invaluable in preventing further deterioration. Current methods for early cognitive impairment detection include face-to-face neuropsychological testing, brain imaging, such as Magnetic Resonance Imaging (MRI), and spinal fluid and blood tests. These methods, however, are often time-consuming and costly. In contrast, blood tests are simpler, easier to administer, and more accessible. Previous studies have identified several blood-based biomarkers that have the potential to signal the presence of neurodegenerative changes [15,16]. Such findings suggest that blood tests hold promise as a tool for assessing cognitive impairment risk and monitoring the effectiveness of preventive treatments [15]. Therefore, the current study is the first to explore this potential correlation using the Periodontal Inflamed Surface Area (PISA) and plasma protein analysis.

The main findings of the present study are: (1) T2DM patients with higher PISA scores tend to be at greater risk for MCI, and (2) moderate significant correlations were observed between inflammation and MCI risk blood-based biomarkers, specifically between PISA and ApoC1, IL-6 and ApoA1, and CRP with ApoA1, C3, and Hemopexin.

No significant difference in HbA1c levels was found between the two groups, suggesting comparable diabetic status. The association between diabetes and increased neurodegeneration risk is well-documented, and our results align with previous studies showing that poor periodontal health may increase the risk of cognitive impairment and dementia [23,24]. A significant difference in MCI risk scores (*p* < 0.05) was observed, with the high PISA group showing a higher MCI risk score (1.37 ± 0.49). This supports the hypothesis that the periodontal inflammation may correlated with systemic proinflammatory mediator levels, thereby contributing to neuroinflammation [25].

The periodontal inflammation has been linked to an increased risk of cognitive impairment; however, it remains unclear whether inflammation initiates the pathological process or primarily accelerates neurodegeneration. Two potential mechanisms have been proposed: an elevated proinflammatory cytokine entering the systemic circulation and the periodontopathogens’ invasion of the brain through peripheral nerves or bloodstream [26,27]. In this study, PISA was used to quantify periodontal inflammation, with scores above 300 mm^2^ indicating moderate-to-severe periodontitis, while lower scores represented healthy-to-mild periodontal conditions [28]. The PISA score, which integrates pocket depth, bleeding, and attachment loss, was chosen as the primary indicator of periodontal inflammation. Since systemic inflammation is a potential pathway linking periodontitis, diabetes mellitus, and cognitive decline, we focused on periodontal inflammation measures to examine their association with systemic inflammatory markers and neurodegeneration. To the best of our knowledge, no standardized categorization of PISA values has been universally established. Given the variability and wide range of PISA scores, we adopted Leira’s categorization to classify the overall periodontal inflammation in this study.

Our results suggest that high PISA scores may reflect a heightened inflammatory burden, contributing to unfavorable plasma biomarker profiles linked to neurodegeneration. Participants with higher PISA scores demonstrated elevated levels of CRP, IL-6, and TNF-α (Table 1), supporting their roles in systemic inflammation and AD pathology. Although Spearman’s correlation analysis did not reveal a significant correlation between PISA scores and individual inflammatory markers, the levels of IL-6, TNF-α, and CRP have been consistently associated with periodontal inflammation in previous research [29,30].

Differences in MCI risk biomarkers were also observed between the two groups. In this study, the lower PISA scores were associated with more favorable levels of ApoA1, TTR, A1BG, Albumin, ApoC1, and A2AP, regardless of statistical significance. These findings align with reports by Inoue (2023) and Uchida (2015), who observed higher levels of these proteins in non-demented controls (NDCs) compared to patients with MCI and AD [15,16]. These biomarkers are involved in processes such as amyloid-β sequestration (ApoA1, TTR), protein nutrition (Albumin), innate immunity (A1BG), lipid metabolism (ApoC1), and coagulation (A2M, A2AP), which may protect against neurodegenerations [15,16]. Significant correlations were identified between several plasma protein biomarkers and inflammatory markers. Elevated IL-6, CRP, and TNF-α levels have been reported in AD patients with diabetes mellitus [31]. TNF-α, in particular, plays a crucial role in neurodegenerative processes by amplifying inflammation, inducing gliosis, demyelination, disruption of the blood–brain barrier (BBB), and cell death [32,33]. Type II diabetes has also been linked to an accelerated cognitive decline rate [34]. Both diabetes mellitus and periodontitis contribute to neurodegeneration through mechanisms that amplify systemic inflammation, impacting wound healing and complement activation, which promotes AD pathogenesis [15,22]. Recent evidence suggests that the contact system’s role in coagulation and fibrinolysis may affect cerebral capillary integrity, a key factor in AD pathology [35]. Elevated levels of IL-6, CRP, and ACT in blood samples have been associated with an increased risk of developing dementia [36].

The combination of ApoA1, TTR, and C3 was found to differentiate MCI from NDCs, reflecting their role in Amyloid-β (Aβ) clearance in healthy individuals [16]. Impaired Aβ production and clearance balance may contribute to the onset of cognitive impairment [37]. CRP’s correlation with ApoA1 and C3 suggests its potential role in Aβ pathogenesis. Bi et al. (2012) speculated that CRP could trigger Aβ production and is elevated during the early stages of AD [38]. Combined cytokines and chemokines, including CRP and IL-6, have been linked to severe cortical atrophy due to enhanced Aβ influx, reduced clearance across the BBB, and increased neural production of Aβ. In this study, CRP was also found to have a significant correlation with hemopexin. However, the bootstrapped 95% confidence interval for the correlation coefficient between these two variables indicates variability in the estimate. The small sample size, high variability within the data, and the non-normal distribution of the data could influence this variability [39].

This study also identified negative correlations between the PISA score and ApoC1. Increasing evidence has shown a correlation between ApoC1 and Alzheimer’s disease; more importantly, ApoC1 is closely associated with cell proliferation, apoptosis, and immune inflammation [40]. However, this connection is influenced by various factors, including genetics, lipid metabolism, and inflammatory responses. Additional research is needed to elucidate the mechanisms by which ApoC1 interacts with systemic inflammation and its role in cognitive decline.

This study had several limitations, including the small sample size and the absence of a non-diabetic control group, which precluded determining the direct effect of diabetic status on biomarker changes. At the outset, the primary aim of the randomized controlled trial (RCT) was to investigate the effect of adjunctive antibiotic periodontal treatment on the risk of mild cognitive impairment (MCI) in patients with diabetes, based on the hypothesis that such treatment would influence systemic inflammation. To minimize variability related to general health status and to control for potential confounding factors, the study population was restricted to diabetic patients, and a non-diabetic control group was not included. However, the present analysis focused on baseline data prior to intervention, and future studies are being considered to include comparisons with non-diabetic patients. Although this study had a relatively small sample size, it was designed as a preliminary randomized controlled trial to evaluate the potential effects of periodontal treatment on MCI risk biomarkers. At the time of planning, no prior studies had investigated similar outcomes using comparable interventions, and thus, no reliable estimates for effect size or standard deviation were available to support a formal sample size calculation. Due to these limitations and resource constraints, we included all available eligible participants in the analysis. While the sample size may not provide sufficient statistical power to detect small to moderate effects, the study was positioned as an early-stage efficacy trial, aiming to generate preliminary evidence for future RCTs. Moreover, no significant difference was observed in the HbA1c levels in both groups. Additionally, the limited number of samples may affect the internal and external validity of the findings. Another point is that this study assessed the risk of MCI primarily through the evaluation of plasma proteins associated with systemic metabolism. In the high PISA group, the average MCI risk score was 1.37, placing participants in the “at-risk of MCI” category based on the MCI Plus screening test. Most patients exhibited a low risk of MCI, which may reflect their well-managed health status. All participants were recruited from an endocrinology clinic, where regular monitoring and management of diabetes is provided. Effective diabetes control likely contributes to healthier lifestyle habits and overall health, potentially mitigating MCI risk scores [41].

Various factors, including age and lifestyle, are likely to influence MCI risk. The average patient ages in the study were 61.60 and 67.11 years for the low and high PISA groups, respectively. Aging, alongside periodontal inflammation, is an independent risk factor for dementia and impairs wound healing, particularly in older adults with diabetes [42]. Neglecting this relationship may compromise periodontal treatment outcomes and exacerbate chronic and systemic inflammation, which could, in turn, negatively impact brain health [6].

Despite these limitations, this preliminary study provides important insights into the potential correlation between periodontal inflammation and MCI risk in diabetic patients. Future research incorporating non-diabetic controls and additional cognitive assessments, such as the Mini-Mental State Examination (MMSE), could enhance the robustness of these findings.

## 5. Conclusions

In conclusion, the systemic inflammation associated with periodontitis and diabetes may increase the risk of neurodegeneration and cognitive impairment. These findings highlight the importance of managing periodontal health as part of a comprehensive strategy to mitigate cognitive decline in patients with diabetes.

## Figures and Tables

**Table 1:** Patient’s characteristics divided by PISA score groups.

Variable	Pisa Score Group		*p*-Value
	≤300 mm^2^ (n = 20)	>300 mm^2^ (n = 9)	
Age	61.60 ± 11.63	67.11 ± 13.54	0.18
Gender			
Male	13 (81.3)	3 (18.8)	
Female	7 (53.8)	6 (46.2)	
No of teeth	25.30 ± 4.25	25.44 ± 4.44	0.87
BMI (kg/m^2^)	25.69 ± 4.06	25.94 ± 2.96	0.90
HbA1c (%)	7.09 ± 0.67	7.32 ± 0.88	0.50
Periodontal Parameters			
PISA (mm^2^)	147.66 ± 82.52	451.98 ± 172.37	<0.01 *
PPD ≥ 4 mm (%)	6.73 ± 4.72	20.60 ± 18.37	0.03 *
BOP (%)	9.84 ± 4.64	28.87 ± 10.82	<0.01 *
Inflammation Parameters			
CRP (mg/dL)	0.09 ± 0.14	0.22 ± 0.31	0.25
IL-6 (pg/mL)	18.59 ± 74.81	26.28 ± 70.19	0.29
TNF-α (pg/mL)	1.68 ± 1.70	2.54 ± 3.63	0.53
MCI Risk Parameters			
ApoA1 (mg/dL)	177.30 ± 38.37	177.10 ± 38.82	1.00
C3 (mg/dL)	120.11 ± 17.47	123.022 ± 16.71	0.98
TTR (mg/dL)	22.10 ± 6.15	20.97 ± 8.01	0.36
ApoC1 (mg/dL)	5.87 ± 1.79	5.80 ± 1.45	0.87
A1BG (mg/dL)	25.75 ± 6.90	23.23 ± 5.77	0.31
Albumin (mg/dL)	5.26 ± 0.64	5.16 ± 0.77	0.66
A2AP (mg/dL)	6.51 ± 0.84	6.40 ± 1.08	0.36
Hemopexin (mg/dL)	107.08 ± 16.82	117.21 ± 24.57	0.50
A2M (mg/dL)	248.03 ± 100.69	281.22 ± 106.50	0.41
MCI Risk score	0.69 ± 0.65	1.37 ± 0.49	0.01 *
No risk—Low risk	15 (75.0)	1 (11.1)	
At risk—High risk	5 (25.0)	8 (88.9)	

* Mann–Whitney U test *p* < 0.05; all data presented in means±SD or n (% of total); PPD: Probing Pocket Depth; BMI: Body Mass Index; BOP: Bleeding on Probing; CRP: C-Reactive Protein; IL-6: Interleukin 6; TNF-α: Tumor Necrosis Factor α; ApoA1: Apolipoprotein A1; C3: Complement C3; TTR: Transthyretin; A1BG: Alpha-1-B-Glycoprotein; ApoC1: Apolipoprotein C1; A2AP: Alpha-2-Antiplasmin; A2M: Alpha-2-Macroglobulin; MCI Risk Score: “No risk” to “Low risk” = 0.00–1.00, “At risk” to “High risk” = 1.01–2.00.

**Table 2:** The risk of MCI according to the PISA scores groups (Fisher’s exact test).

Variable	N	“At risk” to “High risk” of MCI	OR (95% CI)	*p*-Value
PISA Score			24.00 (1.338–30.606)	<0.01 *
≤300 mm^2^ (Ref)	20	5 (25.0%)		
>300 mm^2^	9	8 (88.9%)		

* Fisher’s exact test *p* < 0.05.

**Table 3:** Correlation between periodontal status, inflammation markers, and MCI risk markers.

Variable 1	Variable 2	Spearman’s Correlation (ρ)	*p*-Value	Bootstrapped 95% Confidence Interval
PISA	MCI risk	0.373	0.046	[0.013, 0.657]
	ApoC1	−0.429	0.020	[−0.736, −0.073]
MCI risk	PISA	0.373	0.046	[0.013, 0.657]
	TTR	−0.515	0.004	[−0.777, −0.146]
CRP	ApoA1	−0.423	0.022	[−0.676, −0.074]
	HPX	0.384	0.040	[−0.033, 0.677]
	C3	0.428	0.020	[0.085, 0.708]
IL-6	ApoA1	−0.435	0.018	[−0.695, −0.109]

Spearman’s correlation test *p* < 0.05. The bootstrapped 95% confidence intervals were calculated based on 1000 resamples of the original dataset. PISA: Periodontal Inflamed Surface Area; CRP: C-Reactive Protein; IL-6: Interleukin 6; ApoA1: Apolipoprotein A1; C3: Complement C3; ApoC1: Apolipoprotein C1; HPX: Hemopexin.

## Data Availability

The data presented in this study are available on request from the corresponding author due to patient and hospital’s privacy.

## Abbreviations

The following abbreviations are used in this manuscript:

A1BG	Alpha-1-B-glycoprotein
A2AP	Aplha-2-antiplasmin
A2M	Alpha-2-macroglobulin
Aβ	Amyloid-beta
Alb	Albumin
ApoA1	Apolipoprotein A1
ApoC1	Apolipoprotein C1
AD	Alzheimer’s Disease
BBB	Blood–Brain Barrier
BOP	Bleeding on Probing
C3	Complement component 3
CRP	C-Reactive Protein
HPX	Hemopexin
IL-6	Interleukin 6
MCI	Mild Cognitive Impairment
PPD	Probing Pocket Depth
PISA	Periodontal Inflamed Surface Area
TNF-α	Tumor Necrosis Factor α
TTR	Transthyretin
